# STOP1 and STOP1-like proteins, key transcription factors to cope with acid soil syndrome

**DOI:** 10.3389/fpls.2023.1200139

**Published:** 2023-06-21

**Authors:** Xinbo Li, Yifu Tian

**Affiliations:** ^1^ Hainan Yazhou Bay Seed Lab, Sanya, Hainan, China; ^2^ Center for Advanced Bioindustry Technologies, and Institute of Crop Sciences, Chinese Academy of Agricultural Sciences, Beijing, China

**Keywords:** STOP1 transcription factor, STOP1-like proteins, acid soil syndrome, aluminum toxicity, proton toxicity, plant nutrient, regulatory network

## Abstract

Acid soil syndrome leads to severe yield reductions in various crops worldwide. In addition to low pH and proton stress, this syndrome includes deficiencies of essential salt-based ions, enrichment of toxic metals such as manganese (Mn) and aluminum (Al), and consequent phosphorus (P) fixation. Plants have evolved mechanisms to cope with soil acidity. In particular, STOP1 (Sensitive to proton rhizotoxicity 1) and its homologs are master transcription factors that have been intensively studied in low pH and Al resistance. Recent studies have identified additional functions of STOP1 in coping with other acid soil barriers: STOP1 regulates plant growth under phosphate (Pi) or potassium (K) limitation, promotes nitrate (NO_3_^-^) uptake, confers anoxic tolerance during flooding, and inhibits drought tolerance, suggesting that STOP1 functions as a node for multiple signaling pathways. STOP1 is evolutionarily conserved in a wide range of plant species. This review summarizes the central role of STOP1 and STOP1-like proteins in regulating coexisting stresses in acid soils, outlines the advances in the regulation of STOP1, and highlights the potential of STOP1 and STOP1-like proteins to improve crop production on acid soils.

## Introduction

About 30% of the world’s ice-free land and 50% of the world’s potentially arable lands are acidic (characterized by pH<5.5) (von Uexküll and Mutert, 1995). Approximately 60% of the acid soils occur in tropical or subtropical regions (Kochian et al., 2004), where rainfall is high, leaching is intense, and the soil’s water-holding capacity is low. As a result, acid soils usually have many other factors besides low pH that can impair crop production (Delhaize and Ryan, 1995), including: (a) hypoxia stress caused by submergence and water-logging (Voesenek and Bailey-Serres, 2015); (b) deficiency of soluble basic cations of K, calcium (Ca), and magnesium (Mg) caused by leaching (Krug and Frink, 1983; von Uexküll and Mutert, 1995); (c) dissolving and enrichment of insoluble iron (Fe), Al, and Mn in oxides caused by low pH and hypoxic conditions (Kochian et al., 2004); (d) passivation and deficiency of Pi caused by the fixation of reactive toxic metals (Kochian et al., 2004; Zheng, 2010) together with (e) unbalanced nitrogen nutrition with predominantly ammonium (NH_4_^+^) rather than NO_3_^-^ (Kidd and Proctor, 2001). These factors also accelerate the process of soil acidification.

Acid soils inhibit root elongation and function, affect root water and nutrient uptake, and suppress plant growth (Ma, 2007). As early responsive factors to environmental signals, transcription factors play an essential role in stress resistance. STOP1 is a critical Cys2His2-type zinc finger transcription factor for proton tolerance and Al resistance (Iuchi et al., 2007). Recent studies further demonstrated that STOP1 is involved in regulating nutrient homeostasis and multiple stress tolerance in acid soils. In the post-genomic era, molecular breeding and genetic engineering are effective measures to improve the stress resistance of various crop species. Identification and functional characterization of STOP1 offer promising results for overcoming acid soil syndrome (Iuchi et al., 2007). This review focuses on recent advances in the biological function and regulatory processes of STOP1, which highlights the application of STOP1 and STOP1-like proteins in improving crop resistance to acid soil syndrome.

## Overview of STOP1 and STOP1-like proteins

**Figure 1:**
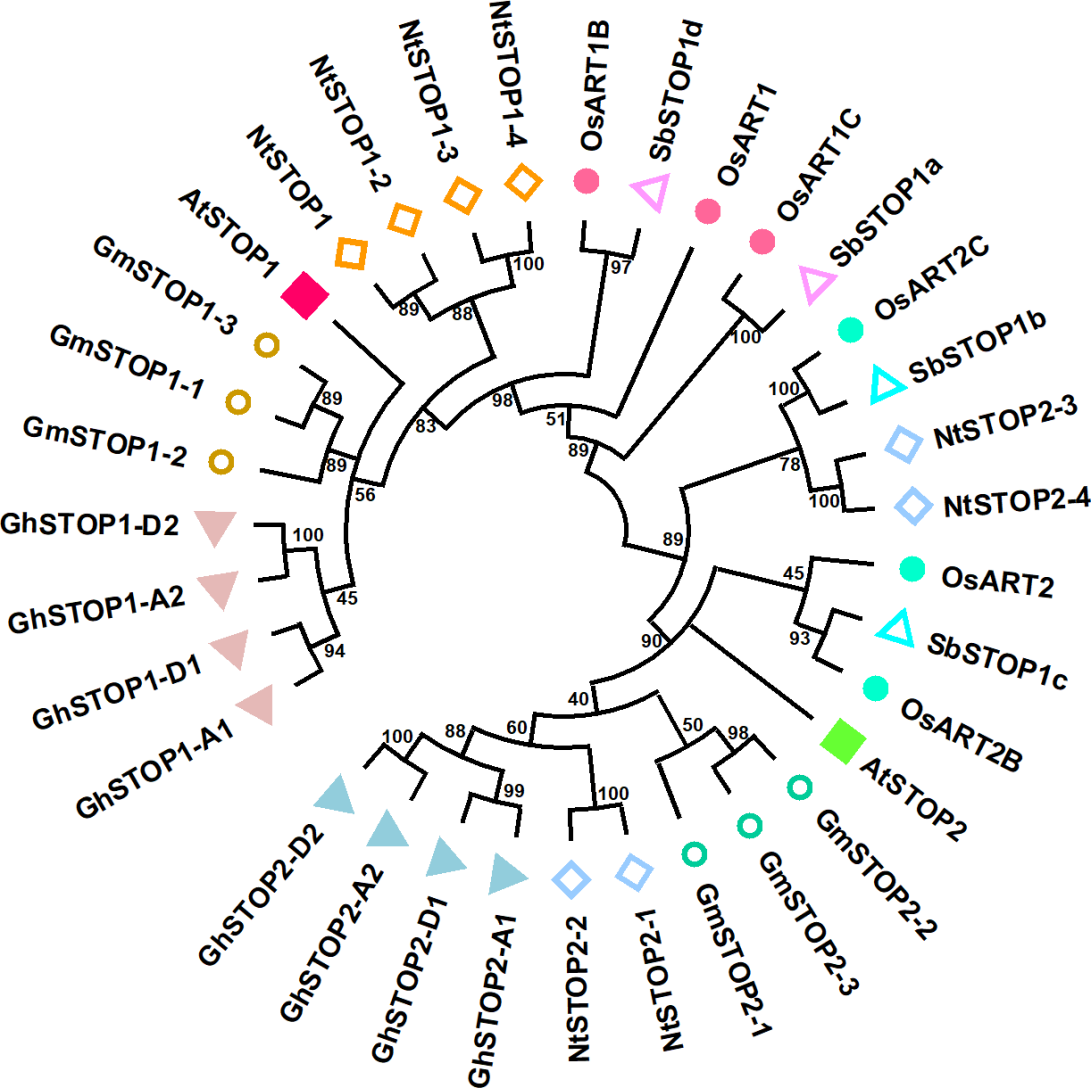
Phylogenetic analysis of STOP1-like proteins in representative crop species. The plant STOP1-like proteins analyzed include representatives from Arabidopsis (*Arabidopsis thaliana* Araport11 data: AtSTOP1, At1G34370; AtSTOP2, At5G22890), soybean (*Glycine max* Wm82 ISU-01 v2.1 data: GmSTOP1-1, Gm10G178500; GmSTOP1-2, Gm16G128700; GmSTOP1-3, Gm20G138900; GmSTOP2-1, Gm13G281700; GmSTOP2-2, Gm11G153300; GmSTOP2-3, Gm12G147100), upland cotton (*Gossypium hirsutum* v3.1 data: GhSTOP1/GhSTOP1-A1, GhA02G060700; GhSTOP1-A2, GhA09G117100; GhSTOP1-D1, GhD02G066100; GhSTOP1-D2, GhD09G114000; GhSTOP2-A1, GhA09G088500; GhSTOP2-D1, GhD09G088200; GhSTOP2-A2, GhA05G086600; GhSTOP2-D2, GhD05G087900), tobacco (*Nicotiana tabacum* v4.5 data: NtSTOP1/NtSTOP1-1, 0000159g0180; NtSTOP1-2, 0009001g0020; NtSTOP1-3, 0000303g0050; NtSTOP1-4, 0004461g0030; NtSTOP2-1, 0000173g0150; NtSTOP2-2 0028281g0010; NtSTOP2-3 0000083g0190; NtSTOP2-4, 0005475g0020), rice (*Oryza sativa* v7.0 data: OsART1, Os12g0170400; OsART1B, Os01g0871200; OsART1C, Os03g0838800; OsART2, Os04g0165200; OsART2B, Os08g0562300; OsART2C, Os02g0572900), sorghum (*Sorghum bicolor* v5.1 data:SbSTOP1a, Sb01G020200; SbSTOP1b, Sb04G188300; SbSTOP1c, Sb07G166000; SbSTOP1d, Sb 03G370700). Evolutionary relationships were inferred from amino acid sequences using the Neighbor-Joining method in MEGA11 (Tamura et al., 2021). The branching topology pattern of the condensed tree is shown under a 40% cut-off.

STOP1 is a C_2_H_2_ zinc finger transcription factor originally identified by forward genetics in Arabidopsis (*Arabidopsis thaliana*). The *stop1* mutant was screened for its low pH sensitivity, and subsequent research showed that this mutant is also hypersensitive to Al stress (Iuchi et al., 2007). STOP1 localizes to the nucleus and up-regulates the expression of many genes involved in low pH tolerance and Al resistance (Sawaki et al., 2009). Recent studies revealed that STOP1 is essential for low-O_2_ (Enomoto et al., 2019), low-Pi (Balzergue et al., 2017; Mora-Macias et al., 2017), low-K (Wang et al., 2021), drought and salt tolerance (Sadhukhan et al., 2019) in Arabidopsis. These findings suggest that STOP1 functions as a central factor in modulating the response to coexisting environmental stresses in acid soils. STOP1 is evolutionarily conserved in a wide range of crops (Garcia-Oliveira et al., 2013; Ohyama et al., 2013; Sawaki et al., 2014; Fan et al., 2015; Huang et al., 2018; Wu et al., 2018; Kundu et al., 2019; Silva-Navas et al., 2021). Homologs of the Arabidopsis STOP1 (AtSTOP1) exist in wheat (*Triticum aestivum*), rice (*Oryza sativa*), soybean (*Glycine max*), tobacco (*Nicotiana tabacum*), sorghum (*Sorghum bicolor*), cotton (*Gossypium hirsutum*), rye (*Secale cereale*), and rice bean (*Vigna umbellata*), etc. Many plant species possess multiple STOP1-like proteins (**Figure 1**). Studies on the biological functions of STOP1-like proteins mainly focused on low pH tolerance and Al resistance. AtSTOP2, the paralog of AtSTOP1, is a physiologically minor isoform that activates the transcription of several AtSTOP1-regulated genes in Arabidopsis (Kobayashi et al., 2014). Knockdown of *AtSTOP2* did not alter proton or Al sensitivity, but overexpression of *AtSTOP2* partially rescued the low pH sensitivity of *Atstop1* (Kobayashi et al., 2014). This is consistent with the fact that *AtSTOP2* has lower expression and functions downstream of *AtSTOP1*, suggesting a possible unequal functional redundancy between them.

Similarly, OsART1 (Al resistance transcription factor 1), a STOP1 homolog in rice, was identified by mutant screening and map-based cloning (Yamaji et al., 2009). As a core transcription factor for Al resistance, OsART1 regulates many Al resistance genes through direct promoter binding and transcription activation (Tsutsui et al., 2011). Although not so sensitive as *Osart1*, the *Osart2* mutants also showed reduced growth under Al^3+^ treatment (Che et al., 2018). However, unlike *AtSTOP1*, mutation of *OsART1* or *OsART2* in rice did not increase sensitivity to proton stress (Yamaji et al., 2009; Che et al., 2018). One possible reason is that OsART1 and OsART2 function redundantly with their homologs in regulating low pH tolerance; since rice has six copies of STOP1-like proteins, neither OsART1 nor OsART2 is the closest homolog to AtSTOP1 (**Figure 1**). Additionally, studies of STOP1 and STOP1-like proteins in other plant species (**Table 1**) showed they have slightly different roles in response to low pH and Al stress (Yamaji et al., 2009; Garcia-Oliveira et al., 2013; Ohyama et al., 2013; Kobayashi et al., 2014; Sawaki et al., 2014; Fan et al., 2015; Che et al., 2018; Daspute et al., 2018; Huang et al., 2018; Wu et al., 2018; Kundu et al., 2019; Silva-Navas et al., 2021). Therefore, future research is needed to evaluate the redundancy between multiple STOP1-like proteins in some plant species, especially in response to low pH, and to investigate whether functional preferences have evolved between them in dealing with specific stresses. The study of the functional preferences of STOP1-like proteins will benefit the extension of STOP1 and STOP1-like proteins to crop genetic improvement.

**Table 1:** Function summary of STOP1 and STOP1-like proteins.

Species	Gene	Host	Method	Low-pH tolerance		Al toxicity tolerance		Other toxic metals	Other stress response	Reference
				pH	Phenotype	Concentration	Phenotype			
*Arabidopsis thaliana*	*AtSTOP1*	*A.thaliana*	Mut/comp	4.7/5.0/5.2	sensitive	2 μM **^#^**	sensitive	not sensitive	low-oxygen, Pi, K, salt, drought	(Iuchi et al., 2007; Balzergue et al., 2017; Mora-Macias et al., 2017; Enomoto et al., 2019; Sadhukhan et al., 2019; Wang et al., 2021)
	*AtSTOP2*	*A.thaliana*	RNAi	4.5/4.7/5.0	not sensitive	2/4 μM **^#^**	not sensitive	ND	ND	(Kobayashi et al., 2014)
		*Atstop1*	OE/comp	4.5/4.7/5.0	slightly rescue	2/4 μM **^#^**	slightly rescue	ND	ND	
*Oryza sativa*	*OsART1*	*O.sativa*	Mut/comp	3.5/4.0/4.5/5.0	not sensitive	10/30/50 μM **^#^**	sensitive	not sensitive	ND	(Yamaji et al., 2009)
	*OsART2*	*O.sativa*	Mut/comp	3.5/4.0/4.5/5.0	not sensitive	10/30/50 μM **^#^**	sensitive	ND	ND	(Che et al., 2018)
*Nicotiana tabacum*	*NtSTOP1*	*N.tabacum*	RNAi	4.7/5.0/5.2	sensitive	2/4 μM **^#^**	sensitive	not sensitive	low-oxygen	(Ohyama et al., 2013; Enomoto et al., 2019)
		*Atstop1*	Comp	4.7	fully rescue	4 μM **^#^**	slightly rescue	ND	ND	
*Lotus japonicus*	*LjSTOP1*	*Atstop1*	Comp	4.7	fully rescue	4 μM **^#^**	slightly rescue	ND	ND	(Ohyama et al., 2013)
*Populus nigra*	*PnSTOP1*	*Atstop1*	Comp	4.7	fully rescue	4 μM **^#^**	slightly rescue	ND	ND	
*Physcomitrella patens*	*PpSTOP1*	*P.patens*	RNAi	4.2	not sensitive	400 μM *	sensitive	ND	ND	
		*Atstop1*	Comp	4.7	fully rescue	4 μM **^#^**	partially rescue	ND	ND	
*Camellia sinensis*	*CsSTOP1*	*Atstop1*	Comp	4.7	fully rescue	4 μM **^#^**	cannot rescue	ND	ND	
*Eucalyptus*	*EguSTOP1*	*Eucalyptus*	RNAi	4.0	sensitive	25 μM *	sensitive	ND	ND	(Sawaki et al., 2014)
		*Atstop1*	Comp	4.7	partially rescue	2 μM **^#^**	cannot rescue	ND	ND	
*Vigna umbellata*	*VuSTOP1*	*Atstop1*	Comp	4.7	partially rescue	2 μM **^#^**	slightly rescue	ND	ND	(Fan et al., 2015)
*Sorghum bicolor*	*SbSTOP1d*	*Atstop1*	Comp	ND	ND	50 μM *	partially rescue	ND	ND	(Huang et al., 2018)
*Secale cereale*	*ScSTOP1*	*Atstop1*	Comp	4.8	fully rescue	300 μM *	fully rescue	ND	low Pi	(Silva-Navas et al., 2021)
*Gossypium hirsutum*	*GhSTOP1*	*G.hirsutum*	RNAi	4.4	sensitive	20 μM **^#^**	sensitive	ND	ND	(Kundu et al., 2019)
*Glycine max*	*GmSTOP1-1*	*Atstop1*	Comp	4.7	partially rescue	2 μM **^#^**	slightly rescue	ND	ND	(Wu et al., 2018)
	*GmSTOP1-2*	*Atstop1*	Comp	4.7	partially rescue	2 μM **^#^**	cannot rescue	ND	ND	
	*GmSTOP1-3*	*Atstop1*	Comp	4.7	partially rescue	2 μM **^#^**	slightly rescue	ND	ND	

Mut, mutation; Comp, complementation; OE, overexpression; ND, not described; **^#^**, hydroponic culture; *, solid medium.

## STOP1 and STOP1-like proteins mediated low pH tolerance

Proton stress is thought to be the proximal cause of poor plant growth in acid soils (Arnon and Johnson, 1942). The primary target of low pH toxicity might be related to the disturbance of the stability in the pectic polysaccharide network (Koyama et al., 2001). In Arabidopsis, AtSTOP1-regulated AtPGIPs (Polygalacturonase inhibitory proteins) inhibit pectin depolymerization in the root cell wall under acidic conditions, maintain the stability of the pectic polysaccharide network, and have a potential role in low pH tolerance (Spadoni et al., 2006).

**Figure 2:**
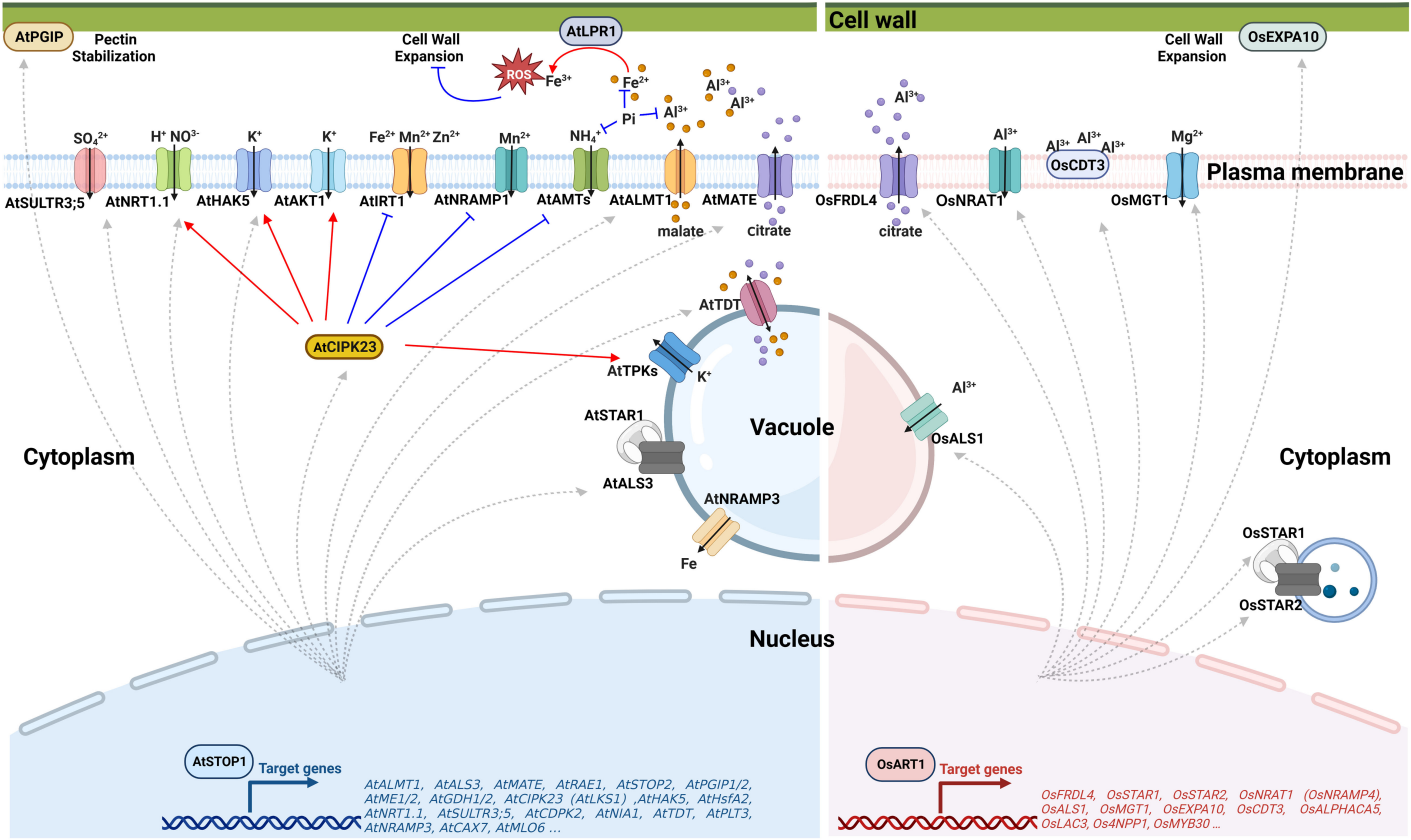
Schematic representation of STOP1/ART1 regulation of Al resistance and nutrient acquisition. Left panel: Under Al stress, AtSTOP1 regulates AtPGIPs to maintain cell walls stability. AtSTOP1 also up-regulates the organic acid transporter coding genes *AtALMT1* and *AtMATE* to increase the secretion of malate and citrate, respectively, which chelate Al in the rhizosphere. For cellular nutrient management, AtSTOP1 enhances Pi bioavailability by regulating malate secretion and Fe-dependent remodeling of root system architecture. AtSTOP1 up-regulates the transcription of *AtHAK5*, *AtNRT1.1*, and *AtSULTR3;5* to facilitate the uptake of K^+^, NO_3_^-^, and SO_4_^2-^, respectively. In addition, AtSTOP1 regulates the activity of AtHAK5, AtNRT1.1, AtAKT1, AtIRT1, AtNRAMP1, AtTPKs, and AtAMTs through AtCIPK23. Right panel: In rice, OsART1 transcriptionally activates *OsFRDL4* to exude citrate, positively regulates *OsSTAR1/2* to maintain cell wall stability, and enhances the Al-induced expression of *OsNRAT1* and *OsALS1* to sequester Al into vacuoles for intracellular detoxification. OsART1 also promotes the expression of the cell wall loosening protein OsEXPA10 and the cysteine-rich peptide OsCDT3 to cope with Al stress. In addition, OsART1 confers Mg uptake through positive regulation of *OsMGT1* transcription to alleviate the Al toxicity. ROS, Reactive oxygen species; STOP1, Sensitive to proton rhizotoxicity 1; ALMT1, Aluminum-activated malate transporter 1; MATE, Multidrug and toxic compound extrusion; TDT, Tonoplast dicarboxylate transporter; PGIPs, Polygalacturonase inhibitory proteins; NRAMP1/3, Natural resistance-associated macrophage protein 1/3; CIPK23/LKS1, CBL-interacting protein kinase 23/Low potassium sensitivity 1; SULTR3;5, Sulfate transporter 3;5; HAK5, High-affinity K^+^ transporter 5; NRT1.1, Nitrate transporter 1.1; AKT1, Arabidopsis K^+^ transporter 1; IRT1, Iron-regulated transporter 1; TPKs, Two-pore K^+^ channels; AMTs, Ammonium transporters; ART1, Al resistance transcription factor 1; FRDL4, Ferric reductase defective-like 4; STAR1/2, Sensitive to Al rhizotoxicity 1/2; EXPA10, Expansin-A10; NRAT1, NRAMP Al transporter 1; ALS1, Al-sensitive 1; MGT1, Magnesium transporter 1. Figure created using BioRender (https://biorender.com/).

The balance of cellular pH is influenced by proton transport across the membrane, H^+^-coupled ion transport, the production or degradation of organic acids, and the uptake and assimilation of nitrogen (Felle, 2001; Britto and Kronzucker, 2005; Reguera et al., 2015; Feng et al., 2020). Activation of several transporters by AtSTOP1 (**Figure 2**) has been reported to be critical for low pH tolerance in Arabidopsis. AtSTOP1 modulates the transcription of *AtHAK5* (High-affinity K^+^ transporter 5) (Sawaki et al., 2009; Nakano et al., 2020), *AtSULTR3;5* (Sulfate transporter 3;5) (Sawaki et al., 2009), H^+^-coupled high-affinity NO_3_^-^ symporter gene *AtNRT1.1* (Nitrate transporter 1.1) (Fang et al., 2016; Ye et al., 2021) and *AtCIPK23* (CBL-interacting protein kinase 23) (Sawaki et al., 2009). AtCIPK23 additionally regulates the activity of AtHAK5 (Ragel et al., 2015; Wang et al., 2021), AtAKT1 (Arabidopsis K^+^ transporter 1) (Li et al., 2006; Xu et al., 2006), AtNRT1.1 (Liu and Tsay, 2003; Leran et al., 2015), and AtAMTs (Ammonium transporters) (Straub et al., 2017; Wang et al., 2021) through phosphorylation to influence ion uptake, overcome rhizosphere acidification and establish a favorable cellular pH. Besides, AtSTOP1 promotes the expression of *AtTDT* (tonoplast dicarboxylate transporter) to increase the concentration of dicarboxylate and, hence, enhance the capacity to produce OH^-^ to regulate the pH homeostasis in the cytosol (Hurth et al., 2005).

Several enzymes involved in metabolic processes that generate or consume protons are also associated with low pH tolerance (Sawaki et al., 2009). Genes encoding malic enzymes (AtME1 and AtME2) that supply pyruvate in the biochemical pH-stat pathway and enzymes (AtGDH1 and AtGAD1) that reduce H^+^ by accumulating GABA in the GABA shunt pathway are transcriptionally regulated by AtSTOP1 (Sakano, 1998; Magneschi and Perata, 2009; Bown and Shelp, 2016). Furthermore, AtSTOP1 transcriptionally regulates its minor isoform AtSTOP2, which co-regulates a subset of AtSTOP1-regulated genes that confer low pH tolerance (Kobayashi et al., 2014).

Although low pH toxicity is the most direct abiotic stress in acid soils, limited studies are still insufficient to fully elucidate the molecular mechanisms by which plants, especially crops, respond to low pH. Further progress is needed to increase our understanding of the diversity and regulating mechanism of low pH resistance genes.

## STOP1 and STOP1-like proteins mediated Al resistance

Al toxicity is one of the most critical factors limiting crop yield in acid soils (Kochian et al., 2015). In acid soils, Al^3+^ ions dissolved from clay minerals enter root cells within 30 minutes and rapidly inhibit root growth within an hour (Lazof et al., 1994; Delhaize and Ryan, 1995). Al-induced exudation of organic acid anions from the roots is the first barrier for plants to cope with Al toxicity. These organic acid anions chelate Al^3+^ to form non-toxic compounds, thereby inhibiting Al entry into the roots (Kochian et al., 2015). In Arabidopsis, AtSTOP1 transcriptionally regulates genes encoding organic acid transporters (**Figure 2**), including *AtALMT1* (Aluminum-activated malate transporter 1) and *AtMATE* (Multidrug and toxic compound extrusion), to increase the secretion of malate and citrate, respectively (Hoekenga et al., 2006; Liu et al., 2009; Sawaki et al., 2009). These organic acids sequestrate toxic Al^3+^ in the rhizosphere, forming a non-toxic complex to reduce plant damage. Similarly, OsART1 activates the transcription of MATE family gene *OsFRDL4* (Ferric reductase defective-like 4) to exude citrate to cope with Al stress in rice (Yokosho et al., 2011). Excess Al^3+^ can still break the barrier of organic acids. The root cell wall is the next site where Al directly contacts and interacts with the plant. The negatively charged groups of pectin and hemicellulose have a high affinity for Al^3+^ and can alleviate Al toxicity by reducing its entry into the root cell (Yang et al., 2008). However, the replacement of Ca^2+^ by Al^3+^ results in a thick and rigid cell wall. Too much Al bound to the cell wall also inhibits root growth and development (Tabuchi and Matsumoto, 2001).

Different strategies have evolved in plants to modify the cell wall in response to Al toxicity. In Arabidopsis, AtSTOP1 up-regulates *AtPGIPs* to strengthen the pectic polysaccharide network in the cell wall under Al stress (Agrahari et al., 2021). In sorghum, SbSTOP1 activates the transcription of a β-1,3-glucanase gene *SbGLU1* to degrade callose and avoid cell wall rigidity (Gao et al., 2019). In rice, OsART1 up-regulates the expression of ABC (ATP binding cassette) transporters OsSTAR1 (Sensitive to Al rhizotoxicity 1) and OsSTAR2 to transport UDP-glucose for cell wall modification, which is required for Al detoxification (Huang et al., 2009). Direct inhibition of *OsMYB30* transcription by OsART1 reduces 4-coumaric acid accumulation, preventing excess Al^3+^ from binding to the cell wall (Gao et al., 2022). OsART1 also promotes the expression of the cell wall loosening protein OsEXPA10 (Expansin-A10) under Al stress, which regulates cell elongation but contributes less to Al tolerance (Che et al., 2016). In addition to the cell wall, Al^3+^ can also be bound to the plasma membrane-anchored cysteine-rich peptide OsCDT3 (Cadmium tolerance 3). OsCDT3 is downstream of OsART1, and it binds Al^3+^ directly to prevent Al^3+^ from entering the root cell, thus alleviating Al toxicity (Xia et al., 2013).

Despite almost 90% of the soluble Al^3+^ in roots being tightly bound to the cell wall (Ma, 2007), a small proportion of toxic Al^3+^ entering the cell can still inhibit root growth (Lazof et al., 1996; Blancaflor et al., 1998; Yamamoto et al., 2002). Once Al enters the root cell, sequestration and storage of Al in the vacuole is an essential mechanism for detoxification (**Figure 2**). OsART1 positively regulates the Al-induced expression of *OsNRAT1* (NRAMP Al transporter 1) and *OsALS1* (Al-sensitive 1) (Yamaji et al., 2009). OsNRAT1 is localized to the plasma membrane, which takes up extracellular Al^3+^ to alleviate cell wall damage (Xia et al., 2010). OsALS1 is a half-size ABC transporter that sequesters the cytoplasmic Al into vacuoles for safe storage (Huang et al., 2012). OsNRAT1 may function cooperatively with OsALS1 to be involved in the intracellular detoxification of Al. In addition, OsART1-regulated OsMGT1 (Magnesium transporter 1) alleviates Al toxicity by increasing intracellular Mg concentration (Chen et al., 2012).

STOP1-like proteins have also been characterized in many plant species (**Table 1**) and show some functional differentiation in response to Al and low pH stress (Ohyama et al., 2013; Sawaki et al., 2014; Fan et al., 2015; Huang et al., 2018; Wu et al., 2018). As orthologous genes with similar functions, the transcription of *OsSTAR1* and *OsALS1* in rice is regulated by OsART1, whereas the expression of *AtSTAR1* and *AtALS1* in Arabidopsis is unaffected by AtSTOP1 (Larsen et al., 2007; Huang et al., 2010). This suggests that different living environments may affect the function of STOP1 and STOP1-like proteins by evolving their preferences for downstream genes. For example, in dryland crops, Al-mediated root exudation of organic acids plays a more important role in Al resistance, whereas rice lives in an aqueous environment that easily disrupts the organic acid barrier (Famoso et al., 2010). Thus, STOP1-like proteins in dryland crops may tend to activate Al resistance genes associated with organic acids secretion, while rice may rely more on cell wall modification and internal detoxification. In addition, overexpression of *AtSTOP2* partially rescued Al resistance and low pH tolerance of *Atstop1* by restoring the expression of AtSTOP1-regulated genes, including *AtPGIP1/2*, *AtALS3*, and *AtMATE*, but not *AtALMT1* (Kobayashi et al., 2014). Whereas in rice, mutation of *OsART2* did not affect the expression of previously identified OsART1-regulated genes (Che et al., 2018). This difference may be due to different experimental approaches, as the knockdown of *AtSTOP2* reduced the expression of *AtPGIP2* and *AtCIPK23* but did not affect the transcription of *AtPGIP1*, *AtALS3* and *AtMATE* (Kobayashi et al., 2014). This difference may also be related to rice having six *STOP1*-like genes that may compensate for each other, with additional copies increasing functional redundancy. In fact, according to RT-qPCR results (Che et al., 2018), some of the potential downstream Al resistance genes identified in *Osart2* are also regulated by OsART1.

Differences in downstream gene sets and regulatory preferences of STOP1-like proteins have been reported in different plants (**Table 2**). In the *Atstop1* complementation assay, STOP1-like proteins showed slight differences in the activation of AtSTOP1 downstream genes (**Table 3**). This suggests there may be a functional differentiation of STOP1-like proteins in different species, or STOP1 partners in Arabidopsis not cooperating well with STOP1-like proteins. Therefore, it is necessary to carry out *in vivo* functional studies in these plants. Despite small differences in the activation of downstream genes by STOP1 and STOP1-like proteins, they remain central factors regulating Al resistance. Further dissection and engineering of STOP1 and STOP1-like proteins have great potential in improving Al resistance in acid soils.

**Table 2:** Identified downstream genes of STOP1 and STOP1-like proteins.

Species	Gene	Method	Decrease expression	Reference
*Arabidopsis thaliana*	*AtSTOP1*	Mut	*AtALMT1, AtALS3, AtMATE, AtRAE1, AtPGIP1/2, AtGDH1/2, AtHsfA2, AtME1/2, AtSTOP2, AtCIPK23, AtHAK5, AtNIA1, AtPLT3, AtSULTR3;5, AtNRT1.1, AtBG3, AtTDT, AtCDPK2, AtCAX7, AtNRAMP3, AtMLO6, AtGRF6, AtCML10, AtESR1, AtPP2C61, AtSAUR54*	(Sawaki et al., 2009; Enomoto et al., 2019; Zhang et al., 2019; Agrahari et al., 2021; Ye et al., 2021)
	*AtSTOP2*	RNAi	*AtPGIP2, AtCIPK23*	(Kobayashi et al., 2014)
*Oryza sativa*	*OsART1*	Mut	*OsFRDL4, OsSTAR1, OsSTAR2, OsNRAT1, OsALS1, OsMGT1, OsEXPA10, OsCDT3, OsLAC3, Os4NPP1, OsALPHACA5*	(Yamaji et al., 2009; Che et al., 2018)
	*OsART2*	Mut	*OsLAC3, Os4NPP1, OsALPHACA5, Os03g0154000*	(Che et al., 2018)
*Nicotiana tabacum*	*NtSTOP1*	RNAi	*NtALS3, NtMATE*	(Ohyama et al., 2013)
*Eucalyptus*	*EguSTOP1*	RNAi	*EguALS3, EguMATE*	(Sawaki et al., 2014)
*Gossypium hirsutum*	*GhSTOP1*	RNAi	*GhMATE, GhALMT1, GhALS3, GhGABAT, GhGAD*	(Kundu et al., 2019)
*Cajanus cajan*	*CcSTOP1*	RNAi	*CcALS3*, *CcMATE1*	(Daspute et al., 2018)

Mut, mutation.

**Table 3:** Expression of STOP1 downstream genes in the *Atstop1* mutant complemented with STOP1-like proteins.

Species	Gene	Method	Fully restore	Partially restore	Cannot restore	Reference
*Arabidopsis thaliana*	*AtSTOP2*	OE/Comp	*AtPGIP1, AtPGIP2, AtALS3, AtMATE*	—	*AtALMT1*	(Kobayashi et al., 2014)
*Eucalyptus*	*EguSTOP1*	Comp	*AtALS3*	*AtCIPK23, AtSTOP2, AtMATE, AtPGIP1*	*AtALMT1*	(Sawaki et al., 2014)
*Vigna umbellata*	*VuSTOP1*	Comp	*AtPGIP1, AtGDH1*	*AtCIPK23, AtSTOP2, AtALS3, AtMATE*	*AtALMT1*	(Fan et al., 2015)
*Nicotiana tabacum*	*NtSTOP1*	Comp	*AtPGIP1, AtPLT3*	*AtCIPK23, AtSTOP2, AtGDH1, AtALS3, AtMATE, AtALMT1*	—	(Ohyama et al., 2013)
*Lotus japonicus*	*LjSTOP1*	Comp	—	*AtPGIP1, AtCIPK23, AtSTOP2, AtALMT1, AtGDH1, AtPLT3*	*AtALS3, AtMATE*	
*Populus nigra*	*PnSTOP1*	Comp	*AtCIPK23*,	*AtPGIP1, AtSTOP2, AtALMT1, AtGDH1, AtPLT3*	*AtALS3, AtMATE*	
*Camellia sinensis*	*CsSTOP1*	Comp	*AtCIPK23*,	*AtPGIP1, AtSTOP2, AtALMT1*	*AtALS3, AtMATE, AtGDH1, AtPLT3*	
*Physcomitrella patens*	*PpSTOP1*	Comp	*AtCIPK23, AtALMT1, AtMATE*	*AtPGIP1, AtSTOP2, AtALS3, AtGDH1, AtPLT3*	—	
*Glycine max*	*GmSTOP1-1*	Comp	—	*AtGDH1/2, AtGABA-T, AtPMI, AtTDT, AtMATE, AtNADP-ME2* (pH and Al^3+^)	—	(Wu et al., 2018)
	*GmSTOP1-2*		—	*AtGDH1/2, AtGABA-T, AtNADP-ME2* (pH)	*AtPMI, AtTDT, AtMATE*, *AtNADP-ME2* (Al^3+^)	
	*GmSTOP1-3*		—	*AtGDH1/2, AtGABA-T, AtPMI, AtTDT, AtMATE, AtNADP-ME2* (pH and Al^3+^)	—	

OE, overexpression; comp, complementation.

## STOP1 and STOP1-like proteins mediated nutrient homeostasis

Nutrient sensing and homeostasis are crucial for plants to adapt to the environment. Soil acidification begins with the loss of salt-based ions, so the acid soils are typically deficient in salt-based ions such as K^+^ and Mg^2+^ (von Uexküll and Mutert, 1995). AtSTOP1 contributes to K uptake by mediating the transcription of *AtHAK5*, which encodes a high-affinity K^+^ transporter (Sawaki et al., 2009). In addition, the AtCIPK23, downstream of AtSTOP1, together with AtCBL1/9 (Calcineurin B-like), enhances K^+^ uptake by phosphorylating the K^+^ transporters AtHAK5 and AtAKT1 (Li et al., 2006; Xu et al., 2006; Ragel et al., 2015; Wang et al., 2021). As a partner of AtCBL2/3, AtCIPK23 also regulates K homeostasis redundantly with AtCIPK3/9/26 through activating tonoplast AtTPK (Two-pore K^+^) channels that promote K^+^ remobilization, which plays a vital role in plant adaptation to K deficiency (Tang et al., 2020). Besides, the AtCBL2/3-CIPK3/9/23/26 module regulates vacuolar Mg storage, thereby influencing Mg homeostasis (Tang et al., 2015). In rice, OsART1 transcriptionally regulates the plasma membrane-localized Mg^2+^ transporter OsMGT1 to promote Mg uptake, especially under Al treatment, thereby increasing cellular Mg content and, on the other hand alleviating Al toxicity (Chen et al., 2012). Apart from the deficiency of salt-based ions, acid soils usually contain excessive levels of metal nutrients such as Fe^2+^, Mn^2+^, and Zn^2+^, which in excess cause phytotoxicity (Kochian et al., 2004). AtCIPK23 phosphorylates the broad-spectrum high-affinity metal transceptor AtIRT1 (Iron-regulated transporter 1) when excess non-iron metals are present and bound to the histidine-rich motif of AtIRT1, which subsequently recruits the E3 ubiquitin ligase AtIDF1, targeting AtIRT1 to the vacuole for its degradation, thus prevent non-iron metal toxicity (Dubeaux et al., 2018). Together with AtCBL1/9, AtCIPK23 interacts with and phosphorylates the high-affinity Mn^2+^ transporter NRAMP1 (Natural resistance-associated macrophage protein 1), promotes its clathrin-mediated endocytosis, reduces its plasma membrane distribution and improves plant tolerance to Mn toxicity (Zhang et al., 2023). In conclusion, AtSTOP1/OsART1 affects the uptake of metals, including K^+^ and Mg^2+^, by controlling the transcription of their transporters directly, and regulates the absorption of K^+^, Mg^2+^, Fe^2+^, Mn^2+^, and Zn^2+^ via CIPK23-mediated phosphorylation.

In addition to affecting the homeostasis of salt-based ions and toxic metals, AtSTOP1 regulates the uptake of non-metallic elements. Under Pi deficiency conditions, AtSTOP1 accumulates in the nucleus and enhances the expression of *AtALMT1* and *AtMATE1* to secrete malate and citrate, respectively. These organic acids desorb Pi from mineral surfaces and dissolve Pi from complexes of Al and Fe oxides, increasing bioavailable Pi concentrations in soil (Kochian et al., 2004). Furthermore, AtSTOP1-promoted malate secretion triggers ROS (reactive oxygen species) production and callose deposition in the presence of the ferroxidases AtLPR1 and AtLPR2 (Low phosphate root 1 and 2), thereby regulating root system architecture, repressing primary root elongation and stimulating lateral root development to efficiently utilize the low mobility Pi in the topsoil (Balzergue et al., 2017; Mora-Macias et al., 2017). During these processes, Fe and Al promote AtSTOP1 accumulation in the nucleus, possibly by inhibiting AtSTOP1 degradation (Godon et al., 2019). Low Pi-induced AtSTOP1 transcriptionally activates *AtALS3*, while AtALS3 interacts with AtSTAR1 to inhibit the nuclear accumulation of AtSTOP1 to prevent AtSTOP1 overactivation (Wang et al., 2019). Low Pi also promotes the expression of *AtAMT1;1* and *AtAMT1;2*, inducing rhizosphere acidification through NH_4_^+^ uptake, which in turn promotes nuclear accumulation of AtSTOP1 to increase soil Pi availability (Tian et al., 2021). In response to the imbalance in nitrogen availability in acidic soils, AtSTOP1-induced AtCIPK23 inhibits the transport activity of AtAMTs through phosphorylation, which alleviates rhizosphere acidification and avoids NH_4_^+^ toxicity (Straub et al., 2017; Wang et al., 2021). AtSTOP1 also controls the NO_3_^-^ uptake by activating *AtNRT1.1* transcription directly, and AtCIPK23 activates AtNRT1.1 through phosphorylation (Ye et al., 2021). Besides, AtSTOP1 positively regulates the expression of *AtSULTR3;5* in the root vasculature, and AtSULTR3;5 is localized to the plasma membrane for sulfate (SO_4_^2-^) uptake and affects the transport of SO_4_^2-^ from root to shoot (Kataoka et al., 2004; Sawaki et al., 2009). Collectively, STOP1 promotes Pi uptake by regulating root system architecture and regulates the uptake of SO_4_^2-^, NH_4_^+^, and NO_3_^-^ by regulating their transporters. It is interesting to note that STOP1 functions as a center for nutrient management under deprivation conditions, controlling nutrient homeostasis other than stress tolerance.

## STOP1 and STOP1-like proteins regulate other stress responses

Approximately 60% of the acid soil occurs in rainfed areas of the tropics or subtropics (Kochian et al., 2004), where plants are sometimes submerged in low (hypoxia) or no oxygen (anoxia) conditions, which are an important abiotic constraint on lowland yields (Zeigler and Puckridge, 1995). In Arabidopsis, *AtSTOP1* transcription is induced by low oxygen. Subsequently, it contributes to low-oxygen tolerance by activating the transcription of *AtGDH1*/*2* and *AtHsfA2* (Heat shock factor A2), and a conserved mechanism that NtSTOP1 involved in hypoxia tolerance has also been identified in tobacco (Enomoto et al., 2019). Furthermore, AtSTOP1 enhances salt tolerance by transcriptionally regulating several salt tolerance genes, including *AtCIPK23*, which negatively regulates drought resistance by maintaining K^+^ transport to maintain stomatal opening (Sadhukhan et al., 2019). AtCIPK23 also directly phosphorylates and activates the S-type anion channels AtSLAC1 (Slow anion channel associated 1) and AtSLAH3 (SLAC1 homolog 3) to regulate the stomatal aperture (Maierhofer et al., 2014). Additionally, AtSTOP1-mediated pH tolerance is involved in the root response of plant-fungal communication between Arabidopsis and *Trichoderma* (Pelagio-Flores et al., 2017).

The *STOP1* homolog in pineapple (*Ananas comosus*) shows a diurnal oscillation expression coinciding with the oscillation of malate concentration in leaves and may be the key circadian oscillator regulating CAM metabolism (Sharma et al., 2017). Mutation of *AtSTOP1* influences the transcript levels of many genes, including *AtPLT3* (Probable polyol transporter 3), *AtCDPK2* (Calcium-dependent protein kinase 2), *AtCAX7* (Calcium exchanger 7), *AtNRAMP3*, *AtNIA1* (Nitrate reductase 1), *AtMLO6* (Mildew resistance locus O 6), *AtGRF6* (Growth regulating factor 6), *AtCML10* (Calmodulin like 10), *AtESR1* (Enhancer of shoot regeneration 1), *AtPP2C61* (Protein phosphatase 2C 61) and *AtSAUR54* (Small auxin upregulated RNA 54) (Sawaki et al., 2009; Sadhukhan et al., 2019). DAP-seq data also showed that AtSTOP1 binds to the promoters of *AtPLT3*, *AtCDPK2*, *AtCAX7*, *AtNRAMP3*, *AtNIA1* and *AtMLO6* (O'Malley et al., 2016). Further studies and more evidence are needed to clarify whether STOP1 participates in other biological processes through these downstream genes.

## Regulation of STOP1 and STOP1-like proteins

**Figure 3:**
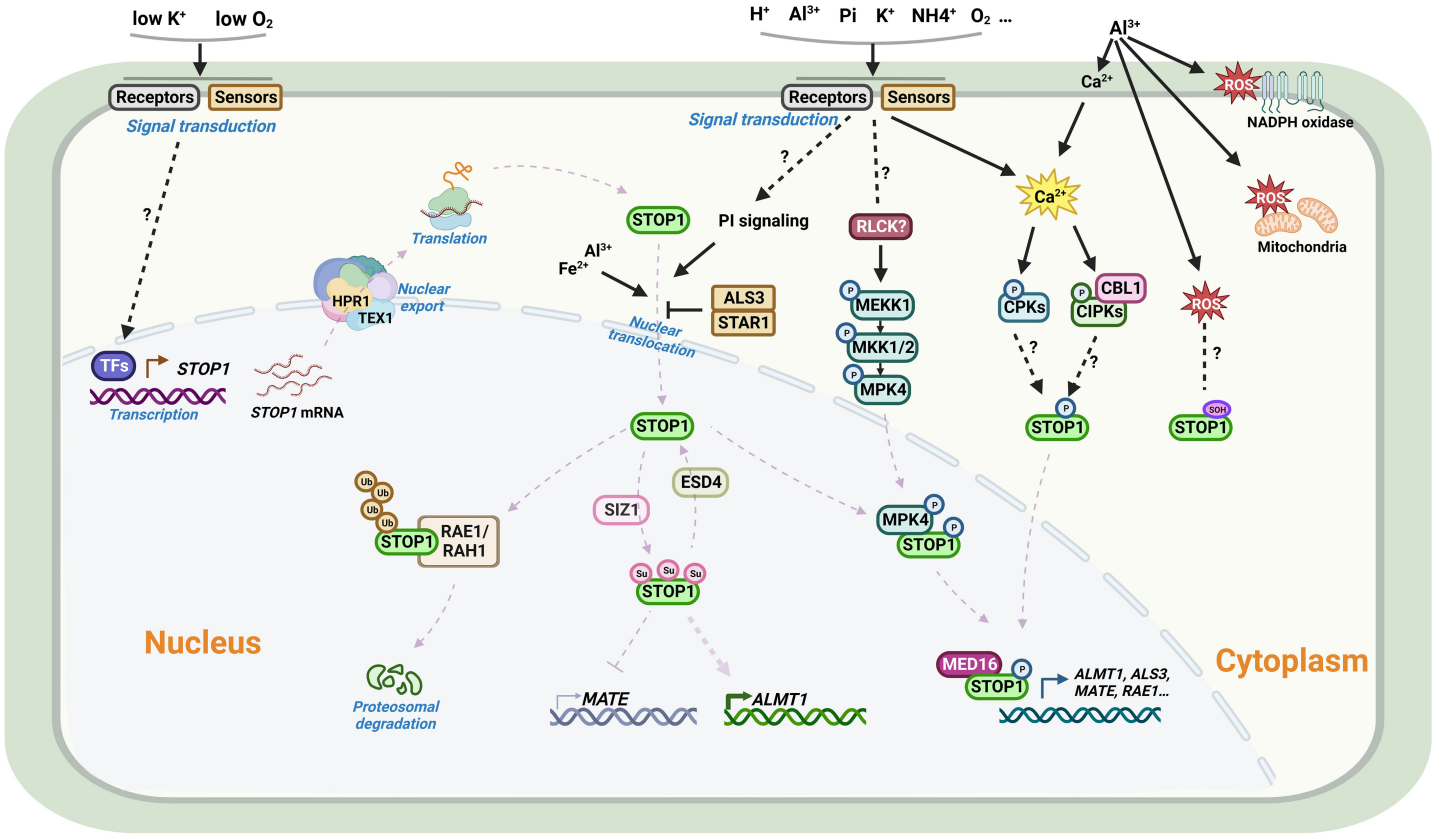
Schematic representation of the regulation of STOP1 in Arabidopsis. From upper left: Under most conditions, *STOP1* is constitutively expressed in plants. Plants can sense hypoxic stress and low K^+^ signals to activate *STOP1* transcription. The mRNA of *STOP1* is processed and transported by the THO/TREX complex on the nuclear envelope. RAE3/HPR1 and RAE2/TEX1 are two key members of the complex, in which HPR1, but not TEX1, affects *STOP1* mRNA export from the nucleus. For the STOP1 protein, in addition to the PI signaling pathway, Al and Fe promote STOP1 nuclear accumulation, whereas ALS3/STAR1 inhibits nuclear STOP1 accumulation. The MEKK1-MKK1/2-MPK4 cascade plays an important role in Al signaling. Al activates the kinase activity of MPK4, which interacts with and phosphorylates STOP1, thereby stabilizing STOP1 by reducing its interaction with RAE1. Al^3+^ releases Ca^2+^ from the cell wall via cation exchange, Ca^2+^ signaling followed by signal transduction may promote STOP1 phosphorylation via CBL/CIPK or CPKs signaling pathways, and Al-induced ROS accumulation may modulate the oxidation of cysteine residues on STOP1. In the nucleus, the F-box proteins RAE1 and RAH1 are components of the SCF-type E3 ligase complex that ubiquitinates STOP1 and facilitates its 26S proteasomal degradation. The SUMO E3 ligase SIZ1 and the SUMO protease RAE5/ESD4 are involved in the SUMOylation and de-SUMOylation modifications of STOP1, regulating its stability and altering its association with different target gene promoters. STOP1 interacts with MED16 to co-activate the transcription of downstream genes. Al, aluminum; Fe, iron; K, Potassium; Pi, Phosphate; Ca, Calcium; H^+^, Proton; NH_4_^+^, Ammonium; O_2_, Oxygen; ROS, Reactive oxygen species; TF, Transcription factor; STOP1, Sensitive to proton rhizotoxicity 1; HPR1, Hyper-Recombination 1; TEX1, Transcription-Export 1; CBL, Calcineurin B-like; CIPK, CBL-interacting protein kinase; CPK, Calcium-dependent protein kinase; MEKK1, MAPK/ERK kinase kinase 1; MKK1/2, MAP kinase kinase 1/2; MPK4, MAP kinase 4; RLCKs, Receptor-like cytoplasmic kinases; PI, phosphatidylinositol; RAE1, Regulation of *AtALMT1* expression; RAH1, RAE1 homolog 1; SUMO, Small ubiquitin-related modifier; SIZ1, SAP and MIZ1 domain-containing ligase 1; ESD4, Early in short days 4; ALS3, Al-sensitive 3; STAR1, Sensitive to Al rhizotoxicity 1; MED16, Mediator 16. Figure created using BioRender (https://biorender.com/).

As a master transcription factor, the activity and protein levels of STOP1 and STOP1-like proteins are regulated by complex mechanisms at multiple levels, including transcriptional regulation, post-transcriptional regulation, and post-translational modifications. Transcript levels of *AtSTOP1* and *OsART1* are not affected by low pH or Al stress, but low K^+^ and hypoxic stress induce *AtSTOP1* transcription in Arabidopsis (Enomoto et al., 2019; Wang et al., 2021). In some plant species, such as sorghum and rice bean, there exists transcriptional regulation of *STOP1*-like genes in response to low pH, Al stress, or cadmium (Cd) stress (**Table 4**). In addition, the mRNA level of the *STOP1* homolog in pineapple showed a more than 3- fold diurnal oscillation within a day, which coincided with the oscillation of malate concentration in leaves (Sharma et al., 2017). Although the transcription of *AtSTOP1* in Arabidopsis is not affected by low pH or Al stress, AtSTOP1 is required for the expression of downstream genes such as *AtALMT1* under Al stress (Iuchi et al., 2007). This suggests the existence of some post-transcriptional regulatory mechanism that promotes the expression of downstream genes by activating AtSTOP1 under Al treatment. Recent studies revealed the mechanisms of *RAE* (Regulation of *AtALMT1* expression) genes in regulating STOP1 at the post-transcriptional level (**Figure 3**). The nuclear membrane localized THO/TREX complex processes *AtSTOP1* mRNA with two key members, AtRAE2/AtTEX1 and AtRAE3/AtHPR1. Mutation of *AtTEX1* or *AtHPR1* decreases the protein level of AtSTOP1 in roots. AtHPR1 affects *AtSTOP1* mRNA export from the nucleus, while AtTEX1 does not (Guo et al., 2020; Zhu et al., 2021).

**Table 4:** Expression induction of *STOP1* and *STOP1*-like genes.

Species	Gene	Expression induced by H^+^	Expression induced by Al^3+^	Expression induced by other stress	Reference
*Arabidopsis thaliana*	*AtSTOP1*	No	No	low K, low O_2_	(Iuchi et al., 2007; Enomoto et al., 2019; Wang et al., 2021)
*Oryza sativa*	*OsART1*	No	No	ND	(Yamaji et al., 2009)
	*OsART2*	slightly	Yes	ND	(Che et al., 2018)
*Vigna umbellata*	*VuSTOP1*	Yes	Yes	Cd	(Fan et al., 2015)
*Secale cereale*	*ScSTOP1*	ND	No	ND	(Silva-Navas et al., 2021)
*Cajanus cajan*	*CcSTOP1*	ND	No	ND	(Daspute et al., 2018)
*Sorghum bicolor*	*SbSTOP1a*	Yes	Yes	Cd	(Huang et al., 2018)
	*SbSTOP1b*	Yes	Yes	Cd	
	*SbSTOP1c*	Yes	Yes	No	
	*SbSTOP1d*	No	Yes	Cd	
*Triticum aestivum*	*TaSTOP1-A*	No	Yes	ND	(Garcia-Oliveira et al., 2013)
	*TaSTOP1-B*	Yes	No	ND	
	*TaSTOP1-D*	No	No	ND	
*Glycine max*	*GmSTOP1-1*	ND	Yes	ND	(Wu et al., 2018)
	*GmSTOP1-2*	ND	Yes	ND	
	*GmSTOP1-3*	ND	Yes	ND	

ND, not described.

The protein level of AtSTOP1 increases after Al treatment, while the E3 ubiquitin ligase AtRAE1 interacts with and ubiquitinates AtSTOP1, promoting its 26S proteasomal degradation (Zhang et al., 2019). As a paralog of AtRAE1, AtRAH1 (RAE1 homolog 1) plays an unequally redundant role in regulating AtSTOP1 stability (Fang et al., 2021b). AtSTOP1 in turn promotes the transcription of *AtRAE1*/*AtRAH1*, forming a negative feedback loop to prevent excessive AtSTOP1 accumulation and over-activation of Al resistance (Zhang et al., 2019; Fang et al., 2021b). Accumulation of AtSTOP1 under Al treatment may result from protein modifications that prevent AtSTOP1 from interacting with AtRAE1/AtRAH1 or inhibit its ubiquitination. Given that inhibitors of PI (phosphatidylinositol) signaling blocked nuclear localization of AtSTOP1 under Al stress, other factors may contribute to the accumulation of AtSTOP1. Under low Pi conditions, Fe and Al-promoted AtSTOP1 accumulation is higher in the *als3* mutants, although the mechanism by which AtALS3/AtSTAR1 inhibits AtSTOP1 accumulation remains unclear (Godon et al., 2019; Wang et al., 2019). In tomato, SlRAE1 is also involved in the ubiquitination and degradation of SlSTOP1, and SlSZP1 (STOP1-interacting zinc finger protein 1) interacts with SlSTOP1 to protect it from degradation by SlRAE1 (Zhang et al., 2022).

Reversible protein phosphorylation affects AtSTOP1-regulated *AtALMT1* transcription and malate secretion (Kobayashi et al., 2007). In rye, a conserved phosphorylatable serine site in ScSTOP1 is vital for activating *ScALMT1* transcription (Silva-Navas et al., 2021). Mutation of *AtCBL1* results in reduced expression of *AtALMT1*, demonstrating that Ca^2+^ signaling may be involved in AtSTOP1 phosphorylation via CBL–CIPK networks (Ligaba-Osena et al., 2017). In addition, the AtMEKK1-AtMKK1/2-AtMPK4 cascade plays a role in AtSTOP1 phosphorylation. Al exposure causes AtMPK4 to phosphorylate AtSTOP1, reducing its interaction with AtRAE1 and thus contributing to the stabilization and accumulation of AtSTOP1 (Zhou et al., 2023). Furthermore, SUMOylation modifications stabilize AtSTOP1, and blocking the SUMOylation of AtSTOP1 reduces AtSTOP1 accumulation. Mutations in the SUMO protease AtRAE5/AtESD4 affect the de-SUMOylation of AtSTOP1 and alter its association with the promoters of different target genes (Fang et al., 2020). Consistently, mutations in the SUMO E3 ligase AtSIZ1 reduce SUMOylation of AtSTOP1 and decrease the protein levels of AtSTOP1 (Fang et al., 2021a; Xu et al., 2021).

In the nucleus, AtSTOP1 interacts with AtMED16 (Mediator 16), a component of the transcriptional co-activation complex, and co-regulates the expression of several downstream genes (Raya-Gonzalez et al., 2021). While AtBZR1 (Brassinazole resistant 1) competitively inhibits the activation of *AtALMT1* expression by AtSTOP1 (Liu et al., 2022). In sorghum, SbSTOP1d is self-interacting and interacts with SbSTOP1b, suggesting that SbSTOP1s may function as homodimers or heterodimers (Huang et al., 2018). The ability that STOP1 to regulate the expression of different genes under different stress conditions suggests that these environmental signals may activate or modify STOP1 in various manners, or there are different kinds of proteins that interact with STOP1 and regulate its promoter binding preferences. Further studies are required to elucidate how these interacting proteins affect the function of STOP1 and STOP1-like proteins, and whether STOP1 and STOP1-like proteins undergo different modifications in response to different environments.

## Concluding remarks and future perspectives

In addition to low pH and proton stress, acidic soils often have many other coexisting factors that impair crop yields. With significant advances in our understanding of the acid soil syndrome, researchers are becoming increasingly aware that although tolerance to low pH is a prerequisite for plant growth in acid soils, STOP1 tolerance is not limited to proton tolerance and Al resistance, but also includes enhanced bioavailability of Pi and other nutrients, as well as tolerance to other limiting factors. As a core transcription factor to cope with acid soil syndrome, STOP1 is a node for the cross-talk of multiple environmental signals. The phenomenon that STOP1 determines many different traits is a classic case of pleiotropy. An evolutionarily effective strategy is using a limited number of genes to perform more functions through combinations of transcriptional regulation, mRNA processing, protein modification, and protein-protein interaction. Because STOP1 has a role in resistance or tolerance to many different stresses, applying STOP1 or STOP1-like proteins in agricultural production is expected to improve crop resistance to acid soil syndrome.

In this review, we summarized the biological functions of STOP1 and STOP1-like proteins, especially in the context of the various constraints of acid soil syndrome. We hope this will provide researchers with insights into exploiting STOP1 and STOP1-like proteins, related signaling components and regulatory networks through molecular breeding and biotechnology to improve crop tolerance to acid soil syndrome, especially those plants that are not well adapted to acid soils, such as alfalfa and soybean. Genome editing is a powerful tool for improving crop varieties. By knocking in cis-elements or high-throughput editing at the *STOP1* promoter region (Shen et al., 2023; Tian et al., 2023), *STOP1* expression can be environmentally induced or constitutively enhanced. The STOP1 protein also can be stabilized by point substitution through base editing of phosphorylation sites (Tian et al., 2022). In addition, the strength of the STOP1 effect can be fine-tuned by modulating potential regulators of STOP1.

Although many regulators of STOP1 under Al stress have been identified, STOP1 and STOP1-like proteins are also regulated by multiple stress signals on acidic soils. Future studies of the regulatory mechanisms of STOP1 may identify more upstream components in the signaling pathway, determine how different receptors sense different upstream signals, and investigate how downstream genes are precisely regulated by STOP1 or STOP1-like proteins. It appears that there are ‘too many’ genes regulated by STOP1 or STOP1-like proteins, and it will also be possible to classify the main process in which STOP1 is involved and to target specific downstream genes to improve a particular trait. Although the function of STOP1 and STOP1-like proteins in Al resistance has been extensively studied in many plant species, it is unclear whether STOP1 and STOP1-like proteins play conserved roles in other biological processes. Further studies are needed to investigate whether other processes regulated by STOP1 are conserved when plants adapt to different living environments. In addition, STOP1 regulates many stress-responsive genes but has a limited effect on certain downstream genes. For some specific genes, transcriptional regulation is not the dominant mode of regulation. Overall, STOP1 contributes more to acid soil tolerance. The other functions of STOP1 are more likely to play a supporting role in dealing with acid soil syndrome. Therefore, we believe that genetic engineering of STOP1 and its homologs is preferred for crops to counteract acidic soils.

## Author contributions

XL conceived the ideas and wrote the draft. YT reviewed and edited the manuscript and the figures. All authors contributed to the article and approved the submitted version.
